# Supplementary material for: Scratching in style: 3D printers as plotters for automated and complex wound-healing assays
Source: iScience. 2025 Nov 26;28(12):114230. doi: 10.1016/j.isci.2025.114230 (PMC12723282; doi:10.1016/j.isci.2025.114230)
Supplement: Document S1. Figure S1 and Table S1 [file mmc1.pdf]

iScience, Volume *28*

Supplemental information

Scratching in style: 3D printers as plotters for automated and complex wound-healing assays

Hanjo Köppe, Magnus G. Richert, Debora Singer, Jorn Köppe, Mattes Köppe, Mladen Tzvetkov, Henry W.S. Schroeder, Sander Bekeschus, and Sandra Bien-Möller

Issue	Example	Cause	Fix
Non-uniform scratch edges due to partial cell retention	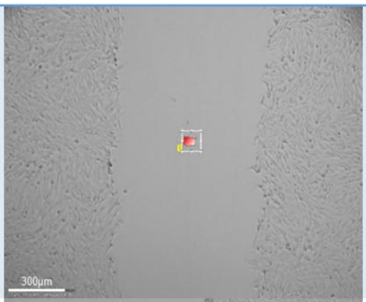	• Cells exhibit strong adhesion to the surface<br>• The pipette tip moves at an insufficiently slow speed<br>• The applied pipette pressure is too low	• Shorten the incubation time<br>• Increase the printing speed<br>• Adjust the z-axis position to a lower value (e.g. move down by 0.5mm)
Removal of large cell sheets	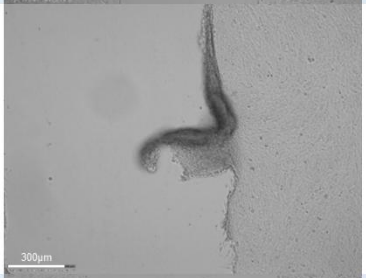	• Cell–cell adhesion is too strong<br>• Adherence of cells to the culture surface is weak	• Reduce the incubation time<br>• Coating of plates with Matrigel or Collagen-1<br>• Increase printing speed to leverage inertia effects
Damaged cell culture plate surface	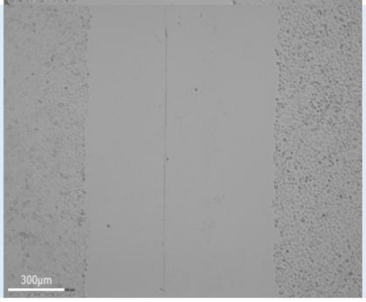	• The pipette tip exerts excessive pressure on the surface<br>• Dust, particles or dried debris are trapped beneath the pipette tip	• Increase the z-axis position (e.g.by + 0.5 mm)<br>• Clean or replace the pipette tip<br>• (Replace the spring-loaded test probe)
No wound		• The pipette tip does not adequately reach the cell surface	• Repeat the calibration process as described in the GitHub documentation<br>• Make sure that the printing bed is properly leveled
The pipette tip makes unintended contact with the plate or nearby structures		• Systematic calibration error<br>• Insufficient safety distance	• Repeat the calibration process as described in the GitHub documentation<br>• Increase the „Tip Offset“ value and input the accurate „Tip Diameter“ for calibration

Figure S1. ASAPR Troubleshooting Guide, related to Figure 1. The most common potential sources of error during ASAPR setup or use are listed. If your specific issue is not included, we encourage to get in touch with us so that we can assist.

	BioTek AutoScratch	Incucyte Wound Maker 96-Tool	SCRATCH (Lin et al.[11])	Custom Scratch Device (Chen et al.[8])	ASAPR
**Source**	Commercial	Commercial	Commercial and costum-built	Costum-built	Commercial and costum-built
**License type**	Closed-source	Closed-source	Open-source	Upon request	Open-source
**Framework**	(-)	(-)	AxiDraw V3	Custom	Most FDM 3D printer/laser engraver/CNC devices
**Cost**	~12.000$	~6.600$	At least ~500$ for the AxiDraw V3	(-)	~15$ (without the 3D printer)
**Production effort**	(-)	(-)	Some 3D printing	Significant (e.g. CNC)	Some 3D printing
**Plate formats**	24- and 96-well	96-well	any	96-well	any
**Number of pins**	4 or 8	96	1	1	1 (upgradable)
**Tip material**	PTFE	PTFE	Adjustable	PTFE	Adjustable
**Spring-loaded tips**	Yes	Yes	No	Yes	Yes
**Self-cleaning**	Yes	Yes	No	No	Yes
**Wound width**	Fix	Fix	Adjustable	Fix	Adjustable
**Complex wound patterns**	No	No	Yes	No	Yes
**CV (wound width)**	1.4-2.1%	(-)	(-)	(-)	1.61%

Table S1. Comparative overview of five scratch-assay techniques, related to introduction. Evaluation of ASAPR relative to two commercially available methods and two established published approaches. (-) indicates “no data”; CNC, Computerized Numerical Control; FDM, Fused Deposition Modeling; CV, coefficient of variation; PTFE, Polytetrafluorethylene. Data for the two commercially available scratch makers were sourced from official product brochures.
